# Luteolin and Quercetin Affect the Cholesterol Absorption Mediated by Epithelial Cholesterol Transporter Niemann–Pick C1-Like 1 in Caco-2 Cells and Rats

**DOI:** 10.1371/journal.pone.0097901

**Published:** 2014-05-23

**Authors:** Mari Nekohashi, Mana Ogawa, Takuo Ogihara, Kyoko Nakazawa, Hisanori Kato, Takumi Misaka, Keiko Abe, Shoko Kobayashi

**Affiliations:** 1 Research Center for Food Safety, Graduate School of Agricultural and Life Sciences, The University of Tokyo, Tokyo, Japan; 2 Faculty of Pharmacy, Takasaki University of Health and Welfare, Takasaki, Gunma, Japan; 3 Corporate Sponsored Research Program “Food for Life,” Organization for Interdisciplinary Research Projects, The University of Tokyo, Tokyo, Japan; 4 Department of Applied Biological Chemistry, Graduate School of Agricultural and Life Sciences, The University of Tokyo, Tokyo, Japan; 5 Kanagawa Academy of Science and Technology Life Science & Environment Research Center 4F C-4, Kawasaki, Kanagawa, Japan; Simon Fraser University, Canada

## Abstract

Niemann–Pick C1-Like 1 (NPC1L1) mediates cholesterol absorption, and ezetimibe is a potent NPC1L1 inhibitor applicable for medication of hypercholesterolemia. Epidemiological studies demonstrated that consumption of polyphenols correlates with a decreased risk for atherosclerosis due to their antioxidant effect. This activity can hardly be attributable to the antioxidant activity only, and we hypothesized that polyphenols inhibit intestinal transport of cholesterol. We elucidated the kinetic parameters of intestinal cholesterol absorption, screened several polyphenols for their ability to specifically inhibit intestinal cholesterol absorption, and determined the inhibitory effects of selected flavonoids *in vitro* and *in vivo*. The concentration-dependent uptake of cholesterol by Caco-2 cells obeyed a monophasic saturation process. This indicates the involvement of an active-passive transport, i.e., NPC1L1. Parameters of cholesterol uptake by Caco-2 cells were as follows: *J*_max_, *K*_t_, and *K*_d_ were 6.89±2.96 19.03±11.58 µM, and 0.11±0.02 pmol/min/mg protein, respectively. Luteolin and quercetin inhibited cholesterol absorption by Caco-2 cells and human embryonic kidney 293T cells expressing NPC1L1. When preincubated Caco-2 cells with luteolin and quercetin before the assay, cholesterol uptake significantly decreased. The inhibitory effects of these flavonoids were maintained for up to 120 min. The level of inhibition and irreversible effects were similar to that of ezetimibe. Serum cholesterol levels significantly decreased more in rats fed both cholesterol and luteolin (or quercetin), than in those observed in the cholesterol feeding group. As quercetin induced a significant decrease in the levels of NPC1L1 mRNA in Caco-2 cells, the *in vivo* inhibitory effect may be due to the expression of NPC1L1. These results suggest that luteolin and quercetin reduce high blood cholesterol levels by specifically inhibiting intestinal cholesterol absorption mediated by NPC1L1.

## Introduction

Hypercholesterolemia is a risk factor for atherosclerosis. Cholesterol as a component of biological membranes as well as a precursor of vitamin D, bile acids, and steroid hormones is nutritionally essential component. However, consuming excess cholesterol affects homeostasis by injuring the inner walls of blood vessels, which interferes with circulation and leads to serious diseases such as cardiac infarction and cerebral apoplexy [1].

Cholesterol homeostasis in humans is mainly balanced by intestinal absorption, endogenous biosynthesis, and biliary/intestinal excretion [2]. Excessive dietary cholesterol intake is one of the major risk factors for hypercholesterolemia and cardiovascular diseases, especially atherosclerosis. Consuming a high calorie- high fat diet induces increased blood cholesterol levels, particularly in people of developed countries [1], [3].

Most patients with hypercholesterolemia are prescribed statins, which inhibits 3-hydroxy-3-methyl-glutaryl-CoA reductase, a component of the mevalonate pathway that affects cholesterol biosynthesis to reduce *de novo* synthesis of cholesterol [4]. Because the mevalonate pathway also induces ubiquinone and dolichol synthesis, statin treatment may affect their concentrations as well as those of other nutrients [5]. Not all patients with hypercholesterolemia are successfully treated only with statins [6].

To address these issues, ezetimibe was developed as a new drug to treat hypercholesterolemia [7], [8]. It specifically inhibits the Niemann–Pick C1-Like 1 (NPC1L1) cholesterol transporter, which is expressed on the brush border membrane of the small intestines of humans, mice, and rats and inhibits cholesterol uptake from the intestine. Although much is known of cholesterol biosynthesis and its regulation, the mechanism of cholesterol absorption has not been well understood until recently. Because cholesterol is hydrophobic, it may be transported across the brush border membrane through passive diffusion. NPC1L1 was identified through the search for ezetimibe molecular targets using a genome-wide bioinformatics screening approach in 2004 [9]. Genetic or pharmaceutical inactivation of NPC1L1 in mice reduces cholesterol absorption by 50% or more and reduces blood cholesterol levels [3], suggesting that NPC1L1 is critical for cholesterol uptake by enterocytes. NPC1L1 is also expressed in the canalicular membrane of human hepatocytes. Because ezetimibe reduces reabsorption of biliary cholesterol [7], [8], it is used to treat hypercholesterolemia. Statins and ezetimibe are prescribed to patients with hypercholesterolemia. There is now a renewed interest in foods and food constituents that are able to inhibit cholesterol absorption.

To mitigate the deleterious consequences of a high cholesterol diet, we screened food constituents able to inhibit cholesterol uptake in the small intestine. Dietary fiber inhibits intestinal cholesterol absorption and causes excretion of cholesterol to the feces. Because the effects are thought to be mediated by their physicochemical properties [10], and because its high dose intake is needed to reduce plasma cholesterol level [11], a highly specific cholesterol absorption inhibitor of food origin is thus required.

Consistent with the French paradox, epidemiological studies show a correlation between consumption of polyphenols and decreased arteriosclerosis risk [12], [13], [14]. Polyphenols have attracted attention because of their potential anti-dyslipidemia activities, strong antioxidant activity, and ability to prevent low-density lipoprotein oxidation in humans [12], [13], [14]. This activity can hardly be attributable to the antioxidant activity only, and we hypothesized that polyphenols would inhibit intestinal transport of cholesterol.

To address this issue, we studied on the mechanisms of polyphenols underlying cholesterol absorption, in particular its kinetics as well as its inhibitor of food origins, providing valuable insights into the prevention and treatment of hypercholesterolemia. Here, we report the identification of polyphenols that inhibit cholesterol uptake in Caco-2 cells and their effects on *in vivo* cholesterol transport.

## Materials and Methods

### Chemicals

The following materials were obtained from commercial sources: Caco-2 cells from Cell Bank, RIKEN BioResource Center (Ibaraki, Japan). [1,2-^3^H(N)]-cholesterol from Perkin Elmer, Inc. (Waltham, MA, USA), ezetimibe from LKT Laboratories, Inc. (St. Paul, MN, USA), cholesterol, sodium taurocholate, lecithin, hesperetin, luteolin, and quercetin from Wako Pure Chemicals (Osaka, Japan); genistein and daidzein from Sigma-Aldrich, Inc. (St. Louis, MO, USA); catechins from Kurita Water Industries Ltd. (Tokyo, Japan); and all the other polyphenols were from Extrasynthese (Genay, France). Cell culture reagents were obtained from Invitrogen (Carlsbad, CA, USA). All the other chemicals used in this study were of reagent grade.

### Caco-2 cells

The cells were seeded in 12-well plates at a density of 1.0×10^5^ cells/well and cultured at 37°C for 7 days in Dulbecco's Modified Eagle Medium (DMEM) containing 10% fetal bovine serum (FBS), 1% nonessential amino acids, 100 U/ml penicillin, 0.1 mg/mL streptomycin, and 50 µg/L gentamycin in a humidified atmosphere containing 5% CO_2_. The medium was replaced every 2 days during culturing.

### Assay of cholesterol uptake by Caco-2 cells

We prepared a cholesterol micellar solution for uptake assays, which contained 1.8 nM [1,2-^3^H(N)]-cholesterol diluted in ethanol, 1 µM cholesterol diluted in ethanol, 4 mM sodium taurocholate, and 100 µM lecithin in Hanks' balanced salt solution (HBSS) (pH 7.4). The mixture was thoroughly vortexed and maintained at 37°C for 2 h before the experiments. On 7 days of culturing, Caco-2 monolayers were washed twice with HBSS (pH 7.4) and incubated in cholesterol micellar solution at 37°C (or 4°C for the temperature dependence assay) for the indicated times in each experiment. At the end of incubation, cells were washed twice with ice-cold HBSS (pH 7.4) and dissolved in 0.1 M ice-cold NaOH. Radioactivity was measured using a liquid scintillation counter (LSC-6100; Aloka, Wallingford, CT, USA).

The cell-to-medium ratio was calculated by dividing the cellular uptake concentration by the cholesterol concentration in the uptake medium. Kinetic parameters for transport activity were established using nonlinear least squares fit of the data using the MULTI program [15] as follows: 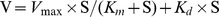
where V, S, *K*_m_, *V*_max_, and *K*_d_ represent the initial uptake rate, substrate concentration, Michaelis constant, maximum uptake rate, and first-order rate constant, respectively.

Ezetimibe (0–20 µM) was preincubated with Caco-2 cells at 37°C for 1 h for the inhibition assay. During the screening, 100 µM solutions of polyphenols diluted in dimethyl sulfoxide (DMSO) were added to the micellar solution. The final total concentration of organic solvent was 1.5%. Before the concentration- and time- dependent inhibition assays, 100 µM luteolin or quercetin were preincubated with Caco-2 cells for 1 h and washed twice with HBSS (pH 7.4). After treatment, the cholesterol micelle was added and its uptake by Caco-2 cells was quantified to measure radioactivity.

### NPC1L1 transfection of HEK cells

NPC1L1 cDNA cloned into pCR-XL-TOPO was purchased from Open Biosystems (Thermo Scientific Abgene, Cambridge, UK) and subcloned into pEF6/V5-HisA (Invitrogen). HEK cells were seeded in 24-well plates at a density of 1.0×10^5^ cells/well in DMEM containing 10% FBS and cultured at 37°C for 24 h in a humidified atmosphere containing 5% CO_2_. HEK cells were transfected with 0.08 µg of DNA using Lipofectamine 2000 (Invitrogen). The medium was changed after 6 h, and cholesterol uptake assays were performed 24 h later. The NPC1L1-and mock-transfected cells (transfected pEF6/V5-HisA) were washed twice with HBSS (pH 7.4), and 20 µM ezetimibe, 100 µM luteolin, or quercetin were added 1 h before incubation at 37°C and 30 min after, 5 mM methyl-β-cyclodextrin was added to deplete intracellular cholesterol. The cells were then washed and incubated with the cholesterol micelle preparation for 2 h to determine cholesterol uptake.

### Animal experiments

Male Wistar rats (CLEA, Tokyo, Japan) aged 7 weeks were divided into four groups (n = 7 or 8 rats each) and orally administered one of the following diets: noncholesterol (NC) with 1% DMSO, high cholesterol (HC) with 1% DMSO, HC with 20 mM luteolin diluted in 1% DMSO at 5 mL/kg body weight (HL), or HC with 20 mM quercetin diluted in 1% DMSO at 5 mL/kg body weight (HQ), respectively. The composition of the diets is shown in Table S1. Rats took diets and water *ad libitum*, and the oral administration was performed twice a day at 10:00 h and 18:00 h. The rats were individually housed at 23°C and 60% humidity under a 12:12 h light–dark cycle for 10 days. We sampled blood from tail veins on days 0, 6, and 9. On day 9, the rats were sacrificed without affiliation, and abdominal blood, liver, and intestinal membranes were collected. Surgery was performed under sodium pentobarbital anesthesia, and all efforts were made to minimize affliction. Serum from tail veins were assayed for cholesterol concentrations using a commercially available detection kit (Cholesterol E-test WAKO). The measurement of cholesterol levels of the serum was performed by ORIENTAL YEAST CO., LTD (Nagahama, Japan). All animal experiments were performed according to the Guidelines for the Care and Use of Animals of The University of Tokyo (approval number: P13-851).

### RNA isolation from Caco-2 cells

Caco-2 cell monolayers (10 cm in diameter) were incubated with 100 µM luteolin and quercetin for 2, 6, and 24 h before washing twice with 10 mL PBS. Then, 500 µL of ISOGEN (Wako) was added to lyse the cells. Total cellular RNA was purified using an RNeasy Mini Kit (Qiagen, Valencia, CA, USA).

### Quantitative real-time polymerase chain reaction (PCR) analysis

qRT-PCR was conducted using SYBR Green EX (Takara Bio, Shiga, Japan) and the Thermal Cycler DICE Real-Time PCR System TP800 (Takara). The primers used for the analysis of each of the mRNAs were as follows: NPC1L1 (forward, 5′-GACCGGCCCAACATCAA-3′, reverse, 5′-CCGCAGAGCTTCTGTGTAATC-3′), ACTB (forward, 5′-GCGTGACATTAAGGAGAAG-3′, reverse, 5′-GAAGGAAGGCTGGAAGAG-3′). The amount of each mRNA was normalized relative to that of ACTB.

### Western blot

Caco-2 cells were incubated for 24 h with 100 µM luteolin and quercetin, washed with ice-cold phosphate-buffered saline, and scraped into 500 µL of lysis buffer (20 mM Tris-HCl, pH 7.4, 100 mM NaCl, 1 mM EDTA, 1% TritonX-100, and 10% glycerol). Protein concentration was determined using the Bradford method with bovine serum albumin as the standard. Each of the lysates (20 µg of protein/well) was subjected to 7.5% sodium dodecyl sulfate-polyacrylamide gel electrophoresis. The protein in gel was electrophoretically transferred to a nitrocellulose membrane, which was then incubated with anti-NPC1L1 antibody diluted 1∶500 (Cell Signaling Technology, Danvers, MA, USA) and secondary antibody (1∶2000) conjugated with horseradish peroxidase. NPC1L1 bands (140 kDa) were detected using an enhanced chemiluminescence advance reagent. The membranes were then stripped and reprobed with the anti-actin antibody (1∶1000) as a loading control.

### Statistical analysis

Each of the data was represented as mean ± standard error. Statistical analyses were performed by repeated-measure one-way or 2-way ANOVA followed by using Dunnett's or Tukey–Kramer tests, with P value of <0.05 considered as significant.

## Results

### Characterization of cholesterol uptake by Caco-2 cells

**Figure 1:**
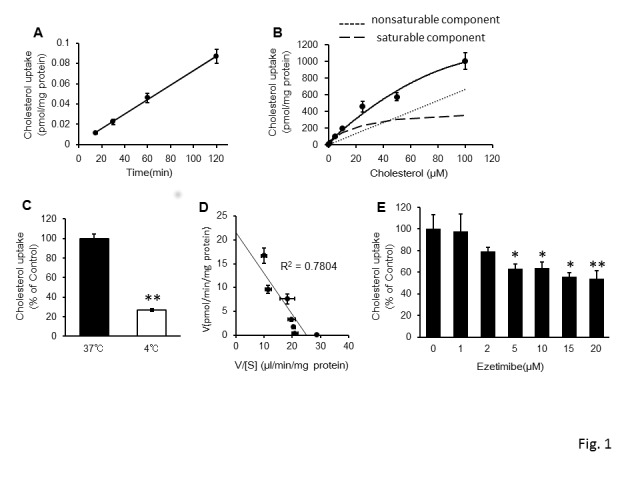
Mechanism of cholesterol uptake by Caco-2 cells (A–D): A, time dependence; B, concentration dependence; C, temperature dependence; D, Eadie–Hofstee plot. The inhibitory effect of ezetimibe preincubation on cholesterol uptake by Caco-2 cells is shown (E). Values are mean ± standard error (n = 3). Statistical analyses were performed using Dunnett's multiple comparison test (*P<0.05; **P<0.01).

The uptake of cholesterol by Caco-2 cells was quantified to define the mechanism of cholesterol uptake thorough the brush border membrane in the small intestine. Because cholesterol uptake by the Caco-2 cell monolayer increased linearly for up to 120 min (Fig. 1A), the following experiments were performed for 60 min.

Fig. 1B shows the concentration dependence of cholesterol uptake. The *J*_max_, *K*_t_, and *K*_d_ values were 6.89±2.96, 19.03±11.58 µM, and 0.11±0.02 pmol/min/mg protein, respectively. Uptake was significantly reduced at 4°C (Fig. 1C). Transformation of the data to an Eadie–Hofstee plot (Fig. 1D) showed a single saturable process with R_2_ value of 0.7804.

Fig. 1E shows that preincubating with ezetimibe inhibited cholesterol uptake mediated by NPC1L1. Ezetimibe treatment reduced intracellular cholesterol concentrations in a dose-dependent manner, and the maximal inhibitory rate was found at approximately 50%.

### Screening of polyphenols that inhibit intestinal cholesterol absorption

**Figure 2:**
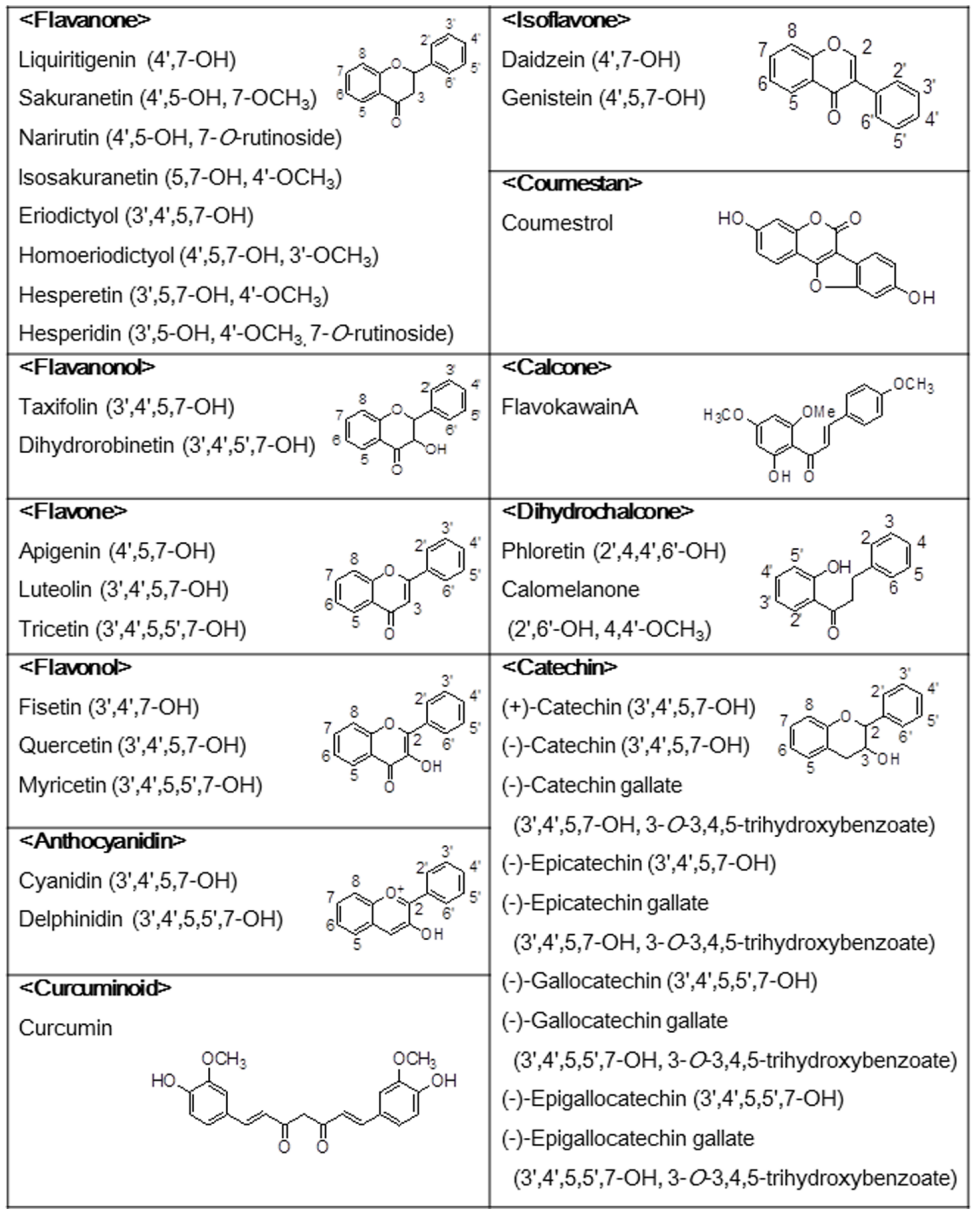
Chemical structure of the screened polyphenols.

**Figure 3:**
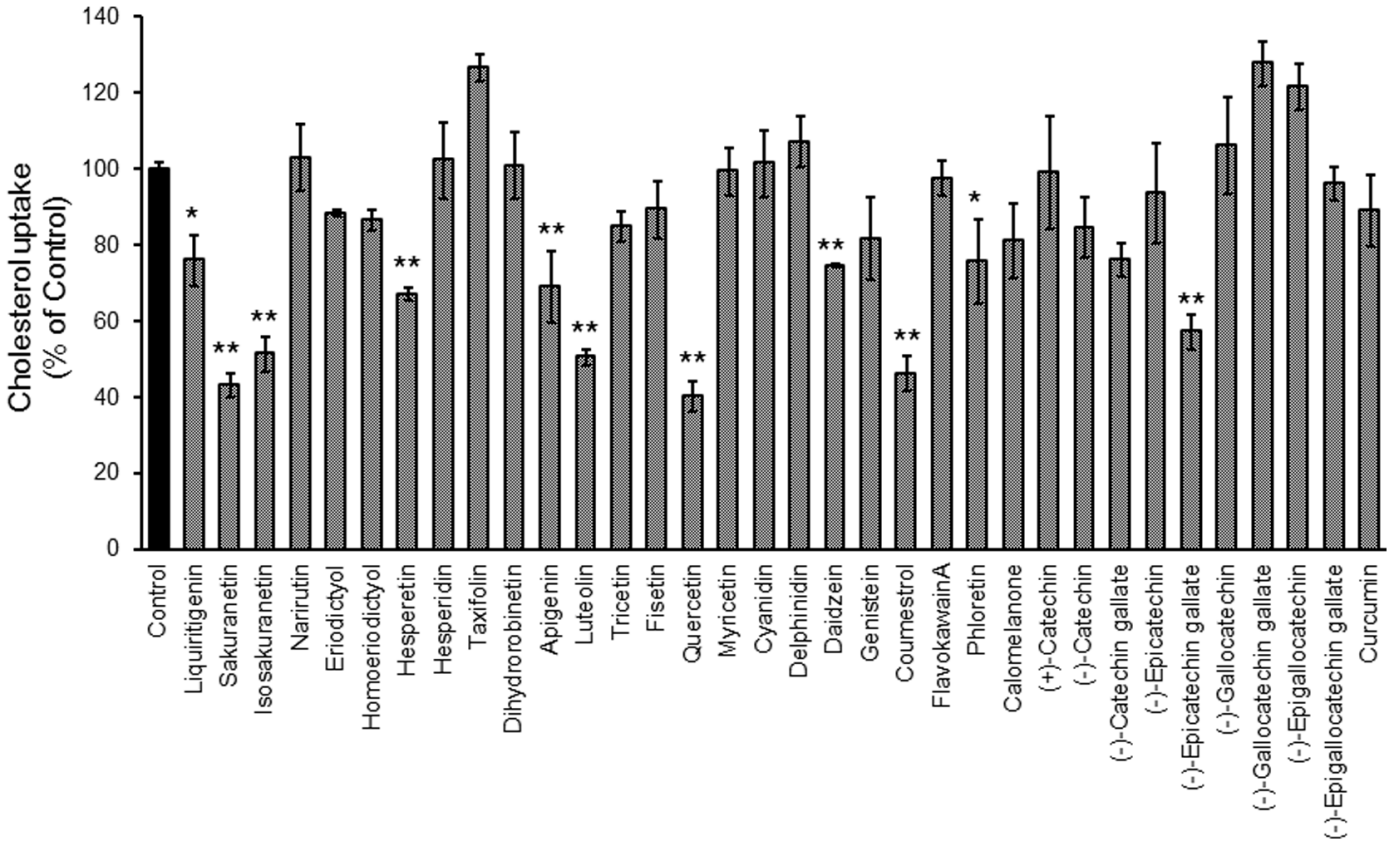
Screening of polyphenols for their inhibitory effects on intestinal cholesterol absorption by Caco-2 cells. Polyphenols (100 µM) were added to the cholesterol micelle solution. The mixture was maintained at 37°C for 1 h and then incubated with Caco-2 cell monolayers. Values are mean ± standard error (n = 3). Statistical analyses were performed using Dunnett's multiple comparison test (*P<0.05; **P<0.01).

We measured the activity of 34 polyphenols for inhibiting cholesterol uptake by Caco-2 cell monolayer cultures (Fig. 2). Each of 11 flavonoids, including liquiritigenin, sakuranetin, isosakuranetin, hesperetin, apigenin, luteolin, quercetin, daidzein, coumestrol, phloretin, and (−)-epicatechin gallate significantly inhibited cholesterol uptake (Fig. 3). Because luteolin and quercetin were potent inhibitors and are present ubiquitously in herbs and edible plants, they were selected for further studies. The relative ratios of cholesterol uptake in the presence of luteolin and quercetin to those of the controls were 40.21%±4.03% and 50.60%±2.08%, respectively.

### Mechanisms by which luteolin and quercetin inhibit cholesterol uptake in Caco-2 and HEK293T cells expressing NPC1L1

**Figure 4:**
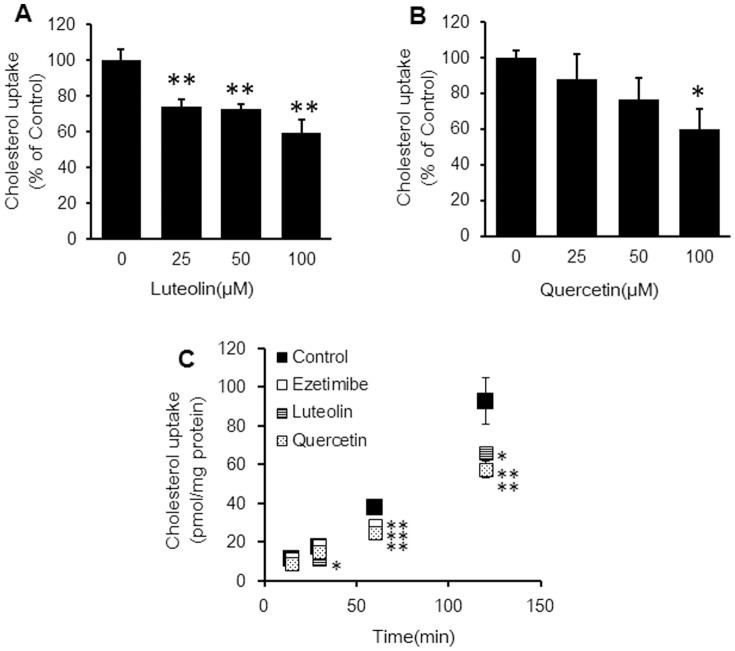
The relationship between the inhibitory effects of cholesterol uptake and concentrations of luteolin (A) and quercetin (B) and stability of the inhibitory effects of luteolin (100 µM), quercetin (100 µM), and ezetimibe (20 µM) (C). Caco-2 cells were preincubated with flavonoids or ezetimibe at 37°C for 1 h, and Caco-2 cells were washed before the cholesterol uptake assay. The cholesterol micelles were added and uptake by Caco-2 cells was quantified to measure radioactivity. Values are mean ± standard error (n = 3). Statistical analyses were performed using Dunnett's multiple comparison test (*P<0.05; **P<0.01).

Caco-2 cells were preincubated with luteolin and quercetin and then washed before the cholesterol uptake assay. Cholesterol uptake through the intestinal membrane decreased in a dose-dependent manner (Fig. 4A, B). Also, the inhibitory effects of luteolin and quercetin were observed 30 and 60 min after, respectively (Fig. 4C). These effects were maintained for up to 120 min, and the plateau of inhibition was similar to the case of ezetimibe.

**Figure 5:**
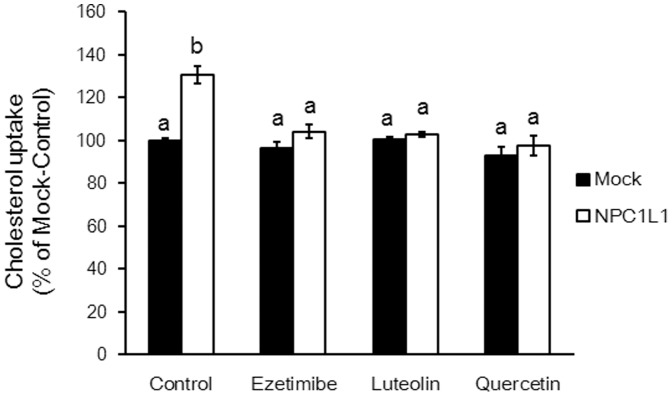
Effects of luteolin (100 µM), quercetin (100 µM), and ezetimibe (20 µM) on cholesterol uptake by HEK293T cells. HEK293T cells were preincubated with flavonoids or ezetimibe at 37°C for 1 h and were washed out before the cholesterol uptake assay. Then, the cholesterol micelles were added, and their uptake was quantified to measure radioactivity. Values are mean ± standard error (n = 3). Statistical analyses were performed using Dunnett's multiple comparison test. Different letters indicate significant differences; P<0.05.

To determine whether luteolin and quercetin inhibited NPC1L1, we tested their effects on cholesterol uptake in HEK293T cells expressing NPC1L1. NPC1L1 transfectants showed significantly increased cholesterol uptake compared with that of mock cells (Fig. 5). However, ezetimibe, luteolin, or quercetin inhibited cholesterol uptake at the same concentrations as in the case of mock transfectants.

### Effects of flavonoids on hypercholesterolemia *in vivo*

**Figure 6:**
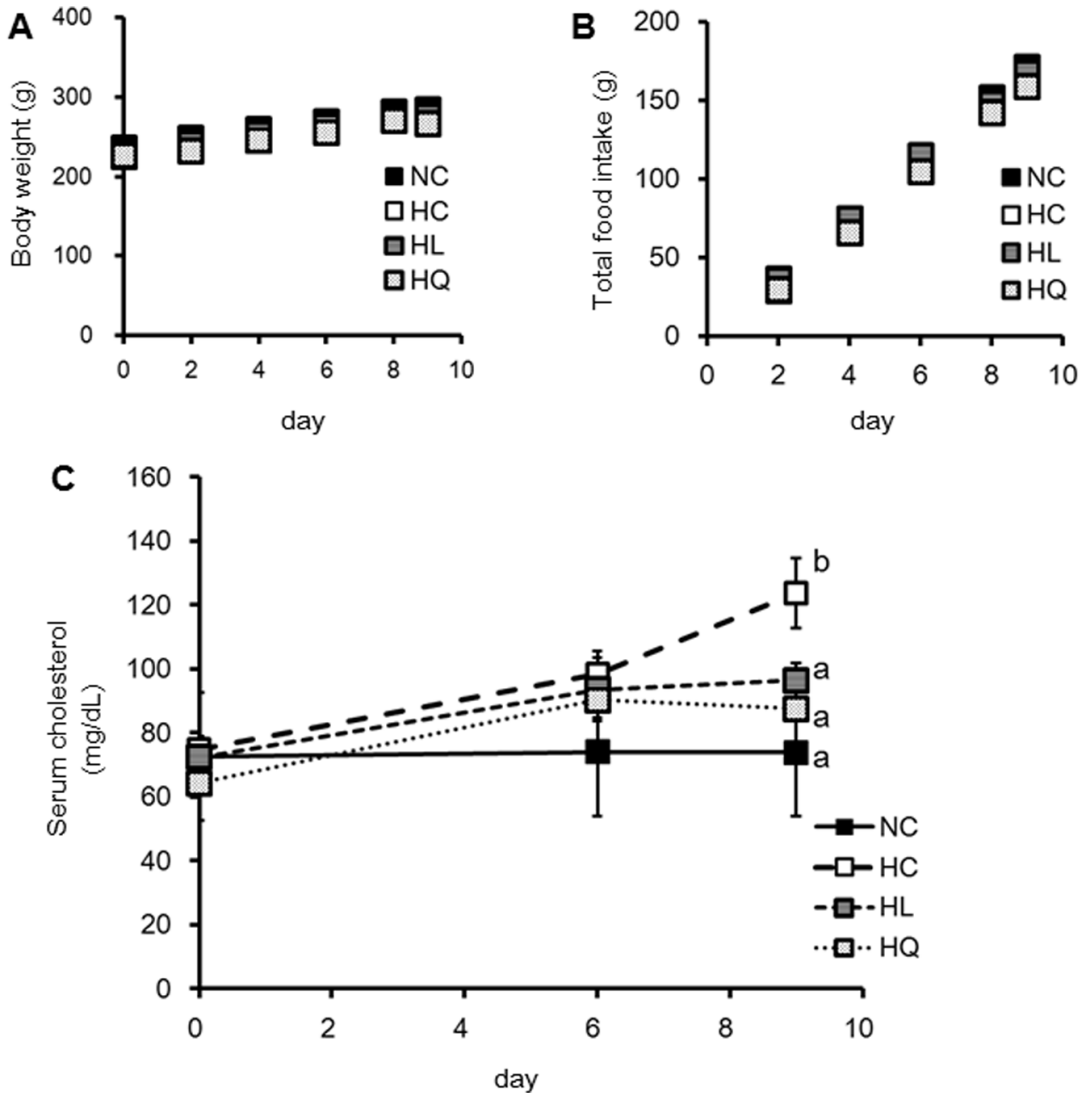
Body weight (A), total food intake (B), and serum concentration of cholesterol (C). Values are mean ± standard error (n = 7 or 8). HC, high cholesterol diet (n = 7); HL, HC with 20 mM luteolin at 5 mL/kg body weight diet (n = 8); HQ, HC with 20 mM quercetin at 5 mL/kg body weight diet (n = 7); NC, noncholesterol diet (n = 7). Statistical analyses were performed using Tukey–Kramer multiple comparison test. Different letters indicate significant differences; P<0.05.

When rats were provided with either a normal AIN93G or cholesterol diet for 9 days with or without flavonoids, no significant difference in food consumption or body weight was observed during feeding (Fig. 6A, B).

Fig. 6C shows the total serum cholesterol levels in rats. The serum cholesterol in the control (non-cholesterol, NC) group was constant over the experimental period, unlike that observed in the cholesterol-fed (high cholesterol, HC) group. However, cholesterol levels in the HL (HC+ luteolin) and HQ (HC+ quercetin) groups were significantly lower than those observed in the HC group.

### Effects of luteolin and quercetin on a decreased in NPC1L1 expression

**Figure 7:**
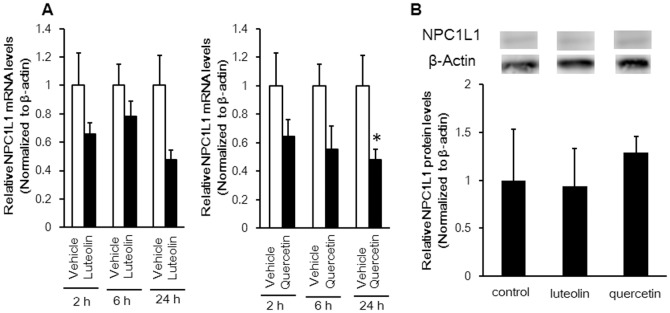
Effects of 100 µM luteolin and quercetin on NPC1L1 mRNA (A) and protein (B) expression in Caco-2 cells. Values are mean ± standard error (n = 3). Statistical analyses were performed using Tukey–Kramer multiple comparison test (*P<0.05; **P<0.01).

Treatment of Caco-2 cells with quercetin for 24 h significantly attenuated the levels of NPC1L1 mRNA (Fig. 7A). The 24-h luteolin treatment decreased the level of mRNA (p = 0.078), whereas levels of the NPC1L1 protein in cells incubated for 24 h with both flavonoids did not change dramatically (Fig. 7B).

## Discussion

The concentration-dependent uptake of cholesterol by Caco-2 cells obeyed a saturation process, indicating an active and passive transport (Fig. 1B). The temperature dependence of transport (Fig. 1C) further supports the prevalence of active transport. Moreover, the Eadie–Hofstee plot was monophasic (Fig. 1D), indicating that cholesterol was incorporated in Caco-2 cells by one transporter. This led us to the conclusion that the transporter was NPC1L1 itself. The present study is the first to report the kinetic parameters of NPC1L1. Furthermore, ezetimibe inhibited cholesterol uptake by Caco-2 cells by 50%, suggesting that the uptake in cells can be chiefly accounted for by NPC1L1. Our finding is in agreement with the results of another independent study [16].

We screened 34 flavonoids (Fig. 2) in Caco-2 cells and found that 11 among them inhibited cholesterol uptake (Fig. 3). Although their activities did not correlate with one another, the structural similarity was that the aglycones, but not glycosides, were active. Luteolin and quercetin, which are ubiquitously present in vegetables [17], significantly inhibited cholesterol uptake and this is a highlight in our research. Certain polyphenols, epigallocatechin gallate, and theaflavins lowered cholesterol absorption and precipitated micellar cholesterol [18], [19]. Cholesterol concentrations in micelles decreased after incubation with 1 mM luteolin and quercetin (Fig. S1). Because the micelle solution was preincubated with the polyphenols during screening, this may include the inhibitory effect of micelle solubility. However, the polyphenol concentration (1 mM) was so high in the micellar solubility assay that the inhibitory effect on the formation of micelles did not play any critical role in Caco-2 cholesterol uptake.

We preincubated Caco-2 cells with flavonoids before the cholesterol uptake assay to determine whether these flavonoids affected intestinal epithelial cells (Fig. 4A, B). As a result, luteolin and quercetin reduced cholesterol uptake in a dose-dependent manner and lowered cholesterol absorption by affecting the intestinal epithelial cells. Furthermore, both luteolin and quercetin showed sustained inhibitory effects similarly to ezetimibe (Fig. 4C) which is known to reduce cholesterol in humans through an irreversible effect on NPCL1 [20]. Thus, these flavonoids could affect NPC1L1 in the same manner as ezetimibe.

We transfected HEK293T cells with an NPC1L1 expression vector to determine whether luteolin and quercetin influenced cholesterol transport mediated by NPC1L1. As shown in Fig. 5, cholesterol uptake increased significantly in the transfectants compared with that in the control mock-transfected cells. However, ezetimibe as well as luteolin and quercetin inhibited cholesterol uptake at a concentration equal to that of the control, suggesting that these flavonoids directly affect cholesterol uptake mediated by NPC1L1. In the *in vivo* experiments, serum cholesterol concentrations significantly decreased in the HL and HQ groups as compared with those in the HC group. When the total-, free-, ester-, LDL-, and HDL- cholesterol were measured form serum of abdominal blood, it was also confirmed that HQ group significantly decreased the total-, free- and LDL- cholesterol level in the serum (data not shown). Luteolin and quercetin are potent natural antioxidants [21], and their function accounts for their anti-arteriosclerosis activity [12], [13], [14]. Here, we showed that this activity can also be attributed to their ability to inhibit cholesterol uptake.

Though luteolin and quercetin inhibited cholesterol uptake mediated by NPC1L1, it is still unclear whether NPC1L1 regulates the systems. Treating Caco-2 cells for 24 h with these flavonoids inhibited NPC1L1 mRNA expression but did not decrease protein concentration (Fig. 7 A, B). The structure of NPC1L1 or the uptake route may have been altered because cholesterol uptake by Caco-2 cells was inhibited by the flavonoids within 1 h. Ezetimibe binding to the middle extracellular domain of NPC1L1 may cause some conformational changes in the NPC1L1 protein to disturb NPC1L1 and cholesterol interactions and ultimately inhibiting cholesterol-induced NPC1L1 endocytosis [2], [7]. One possibility is that these flavonoids also bind to the extracellular domain of NPC1L1 to result in conformational changes of the NPC1L1 protein, thereby inhibiting NPC1L1. In contrast, the N terminus of NPC1L1 helps to move extracellular cholesterol to the membrane-localized sterol-sensing domain (SSD) region to create a raft-like plasma membrane microdomain [3]. Green tea catechins inhibit ileal bile acid transporter activity by suppressing plasma membrane cholesterol [22]. If flavonoids suppress cholesterol in the microdomain, its composition may change, with the result that NPC1L1 transport would be inhibited indirectly. Anyway, the flavonoid treatment was so short that the inhibitory effect on NPC1L1 expression did not occur under this experimental condition. In contrast, because the rats were fed flavanoids for 10 days (Fig. 6), the *in vivo* inhibitory effect *may* be included in the NPC1L1expression.

Our results show for the first time that quercetin inhibited NPC1L1 mRNA expression, albeit by an unknown mechanism. Nuclear receptors, including peroxisome proliferator activated receptor (PPAR)α, PPARδ, liver X receptor, retinoid X receptor, and sterol regulatory element-binding protein 2 are implicated in the regulation of intestinal cholesterol absorption [3], [23], but it is unknown whether they function by modulating intestinal NPC1L1 expression directly. Thus, whether luteolin and quercetin act through a nuclear receptor to inhibit NPC1L1 mRNA expression requires further investigation.

When NPC1L1 SSD detects an increase in cholesterol in the microdomain, the NPC1L1–cholesterol complex is internalized through clathrin-mediated endocytosis [3], [7], [24]. The motifs of two potential YXXØ tetrapeptides facilitate clathrin-mediated endocytosis with the adaptor protein (AP) complex AP2 [3], [7], [24]. Recently, a novel endocytic motif, YVNXXF, was identified in the cytoplasmic C-terminal tail of NPC1L1 [25]. Flotillins also mediate cholesterol endocytosis [26].

Ezetimibe binds NPC1L1 and blocks its cholesterol absorption. However, ezetimibe also prevents interaction of NPC1L1 with clathrin/AP2 coated vesicles [27]. Chlorpromazine (CPZ, a clathrin-mediated endocytosis inhibitor) reduced cholesterol uptake in Caco-2 cell monolayers at 50 µM (Fig. S2). When 50 µM luteolin and quercetin with 50 µM CPZ were added to the Caco-2 cell monolayers, their inhibitory activities were higher than those of CPZ alone (Fig. S2). This result suggests that these flavonoids inhibit the connection between cholesterol, NPC1L1, and clathrin-mediated endocytosis.

Our data suggests that flavonoids inhibit cholesterol uptake through multiple mechanisms. For example, they may be involved in 1) inhibiting the binding of cholesterol to NPC1L1; 2) inhibiting clathrin-mediated endocytosis of NPC1L1 mediated by SSD, YXXØ motifs, AP2, flotillin and YVNXXF motif; and 3) altering the microdomain component of intestinal epithelial cells. Further studies will be needed to clarify this issue.

Rodents express NPC1L1 only in the intestine, whereas humans express it in the liver and intestine [2], [3], [7]. NPC1L1 localizes in the bile canalicular membrane of the liver and contributes to absorption of cholesterol from bile acids. Hepatic overexpression of human NPC1L1 dramatically reduces biliary cholesterol levels. Consequently, flavonoids can inhibit NPC1L1 function in the intestine and liver of humans.

In conclusion, luteolin and quercetin reduce high blood cholesterol levels by inhibiting the intestinal cholesterol absorption mediated by NPC1L1. This is a newly found polyphenol function known as the French paradox.

## Supporting Information

Figure S1 Inhibitory effects of luteolin and quercetin on micellar solubility of cholesterol. The effects of polyphenols on micellar solubility of cholesterol were assayed according to the method of Ikeda et al. [18], [19]. A bile salt micellar solution containing 4 mM sodium taurocholate, 0.1 mM egg yolk phosphatidylcholine (Sigma-Aldrich), 0.5 mM cholesterol, 0.3 nM [1,2-^3^H(N)]-cholesterol, and 1% (v/v) methanol was prepared by vortexing and stored at 37°C for at least 24 h. Polyphenols (final concentration: 1 mM each) were added to the micellar solution (100 µL) and maintained at 37°C for 1 h. The solution was passed through a 0.22-µm PVDF membrane filter (Ultrafree, Millipore), and the concentration of radioactive cholesterol in the filtrate was measured using a scintillation counter. Hesperidin, which did not significantly affect cholesterol uptake (Fig. 3), was used as a negative control. Values are mean ± standard error (n = 3). Statistical analyses were performed as in Fig. 1.
(TIF)

Figure S2 Inhibitory effects of simultaneously adding chlorpromazine (CPZ) with luteolin or quercetin. The Caco-2 cells were incubated in 50 µM CPZ (a clathrin-mediated endocytosis inhibitor) with or without 50 µM luteolin or quercetin at 37°C for 1 h, washed twice with HBSS (pH 7.4). The cholesterol micelle was then added, and uptake by Caco-2 cells was quantified. Values are mean ± standard error (n = 3). Statistical analyses were performed as described in the legend to Fig 5.
(TIF)

Table S1 Composition of diets and oral administration of flavonoids.
(DOCX)

## References

[1] Gadgil MD, Anderson CA, Kandula NR, Kanaya AM (2013) Dietary Patterns in Asian Indians in the United States: An Analysis of the Metabolic Syndrome and Atherosclerosis in South Asians Living in America Study. J Acad Nutr Diet. 10.1016/j.jand.2013.09.021. PubMed: 24295929 PMC3947024 24295929

[2] Davis HR Jr, Altmann SW (2009) Niemann-Pick C1 Like 1 (NPC1L1) an intestinal sterol transporter. Biochim Biophys Acta 1791: 679–683 10.1016/j.bbalip.2009.01.002. PubMed: 19272334 19272334

[3] Jia L, Betters JL, Yu L (2011) Niemann-pick C1-like 1 (NPC1L1) protein in intestinal and hepatic cholesterol transport. Annu Rev Physiol 73: 239–259 10.1146/annurev-physiol-012110-142233. PubMed: 20809793 20809793 PMC3965667

[4] Istvan ES, Deisenhofer J (2001) Structural mechanism for statin inhibition of HMG-CoA reductase. Science 292: 1160–1164 10.1126/science.1059344. PubMed: 11349148 11349148

[5] Wyman M, Leonard M, Morledge T (2010) Coenzyme Q10: a therapy for hypertension and statin-induced myalgia? Cleve CliN J Med 77: 435–442 10.3949/ccjm.77a.09078. PubMed: 20601617 20601617

[6] Devroey D, Radermecker RP, Van der Schueren BJ, Torbeyns B, Jaken RJ (2013) Prevalence of persistent lipid abnormalities in statin-treated patients: Belgian results of the Dyslipidaemia International Study (DYSIS). Int J Clin Pract 68: 180–187 10.1111/ijcp.12315. PubMed: 24308644 24308644 PMC4265243

[7] Betters JL, Yu L (2010) NPC1L1 and cholesterol transport. Febs Lett 584: 2740–2747 10.1016/j.febslet.2010.03.030. PubMed: 20307540 20307540 PMC2909875

[8] Weinglass AB, Kohler M, Schulte U, Liu J, Nketiah EO, et al (2008) Extracellular loop C of NPC1L1 is important for binding to ezetimibe. Proc Natl Acad Sci U S A 105: 11140–11145 10.1073/pnas.0800936105. PubMed: 18682566 18682566 PMC2516253

[9] Altmann SW, Davis HR Jr, Zhu LJ, Yao X, Hoos LM, et al (2004) Niemann-Pick C1 Like 1 protein is critical for intestinal cholesterol absorption. Science 303: 1201–1204 10.1126/science.1093131. PubMed: 14976318 14976318

[10] Schneeman BO. (1999) Fiber, inulin and oligofructose: similarities and differences J Nutr 129:1424S–1427S. PubMed: 10395611 10395611 10.1093/jn/129.7.1424S

[11] Anderson JW, Baird P, Davis RH Jr, Ferreri S, Knudtson M, et al (2009) Health benefits of dietary fiber. Nutr Rev 67: 188–205 10.1111/j.1753-4887.2009.00189.x. PubMed: 19335713 19335713

[12] Opie LH, Lecour S (2007) The red wine hypothesis: from concepts to protective signalling molecules. Eur Heart J 28: 1683–1693 10.1093/eurheartj/ehm149. PubMed: 17561496 17561496

[13] Zern TL, Fernandez ML (2005) Cardioprotective effects of dietary polyphenols. J Nutr 135:2291–2294. PubMed: 16177184 16177184 10.1093/jn/135.10.2291

[14] Rasmussen SE, Frederiksen H, Struntze Krogholm K, Poulsen L (2005) Dietary proanthocyanidins: occurrence, dietary intake, bioavailability, and protection against cardiovascular disease. Mol Nutr Food Res 49: 159–174 10.1002/mnfr.200400082. PubMed: 15635686 15635686

[15] Yamaoka K, Tanigawara Y, Nakagawa T, Uno T (1981) A pharmacokinetic analysis program (multi) for microcomputer. J Pharmacobiodyn 4:879–885. PubMed: 7328489. 7328489 10.1248/bpb1978.4.879

[16] Feng D, Ohlsson L, Duan RD (2010) Curcumin inhibits cholesterol uptake in Caco-2 cells by down-regulation of NPC1L1 expression. Lipids Health Dis 9: 40–45 10.1186/1476-511X-9-40. PubMed: 20403165 20403165 PMC2865464

[17] Ross JA, Kasum CM (2002) Dietary flavonoids: bioavailability, metabolic effects, and safety. Annu Rev Nutr 22: 19–34 10.1146/annurev.nutr.22.111401.144957. PubMed: 12055336 12055336

[18] Ikeda I, Yamahira T, Kato M, Ishikawa A (2010) Black-tea polyphenols decrease micellar solubility of cholesterol in vitro and intestinal absorption of cholesterol in rats. J Agric Food Chem 58: 8591–8595 10.1021/jf1015285. PubMed: 20681647 20681647

[19] Ikeda I, Imasato Y, Sasaki E, Nakayama M, Nagao H, et al (1992) Tea catechins decrease micellar solubility and intestinal absorption of cholesterol in rats. Biochim Biophys Acta 1127:141–146. doi.org/10.1016/0005-2760(92)90269-2. PubMed: 1643098 1643098 10.1016/0005-2760(92)90269-2

[20] Miura S, Saku K (2008) Ezetimibe, a selective inhibitor of the transport of cholesterol. Intern Med 47: 1165–1170 10.2169/internalmedicine.47.1099.. PubMed: 18591835 18591835

[21] Guerra-Araiza C, Álvarez-Mejía AL, Sánchez-Torres S, Farfan-García E, Mondragón-Lozano R, et al (2013) Effect of natural exogenous antioxidants on aging and on neurodegenerative diseases. Free Radic Res 47: 451–462 10.3109/10715762.2013.795649. PubMed: 23594291 23594291

[22] Annaba F, Kumar P, Dudeja AK, Saksena S, Gill RK, et al (2010) Green tea catechin EGCG inhibits ileal apical sodium bile acid transporter ASBT. Am J Physiol Gastrointest Liver Physiol 298: G467–473 10.1152/ajpgi.00360.2009. PubMed: 20056894 20056894 PMC2838517

[23] Iwayanagi Y, Takada T, Tomura F, Yamanashi Y, Terada T, et al (2011) Human NPC1L1 expression is positively regulated by PPARα. Pharm Res 28: 405–412 10.1007/s11095-010-0294-4. PubMed: 20953676 20953676

[24] Zhang JH, Ge L, Qi W, Zhang L, Miao HH, et al (2011) The N-terminal domain of NPC1L1 protein binds cholesterol and plays essential roles in cholesterol uptake. J Biol Chem 286: 25088–25097 10.1074/jbc.M111.244475. PubMed: 21602275 21602275 PMC3137082

[25] Li PS, Fu ZY, Zhang YY, Zhang JH, Xu CQ, et al (2014) The clathrin adaptor Numb regulates intestinal cholesterol absorption through dynamic interaction with NPC1L1. Nat Med 20: 80–86 10.1038/nm.3417 24336247

[26] Ge L, Qi W, Wang LJ, Miao HH, Qu YX, et al (2011) Flotillins play an essential role in Niemann-Pick C1-like 1-mediated cholesterol uptake. Proc Natl Acad Sci U S A 108: 551–556 10.1073/pnas.1014434108. PubMed: 21187433 21187433 PMC3021008

[27] Ge L, Wang J, Qi W, Miao HH, Cao J, et al (2008) The cholesterol absorption inhibitor ezetimibe acts by blocking the sterol-induced internalization of NPC1L1. Cell Metab 7: 508–519 10.1016/j.cmet.2008.04.001. PubMed: 17681147 18522832

