# Supplementary figures and images for: Luteolin and Quercetin Affect the Cholesterol Absorption Mediated by Epithelial Cholesterol Transporter Niemann–Pick C1-Like 1 in Caco-2 Cells and Rats
Source: PLoS One. 2014 May 23;9(5):e97901. doi: 10.1371/journal.pone.0097901 (PMC4032257; doi:10.1371/journal.pone.0097901)

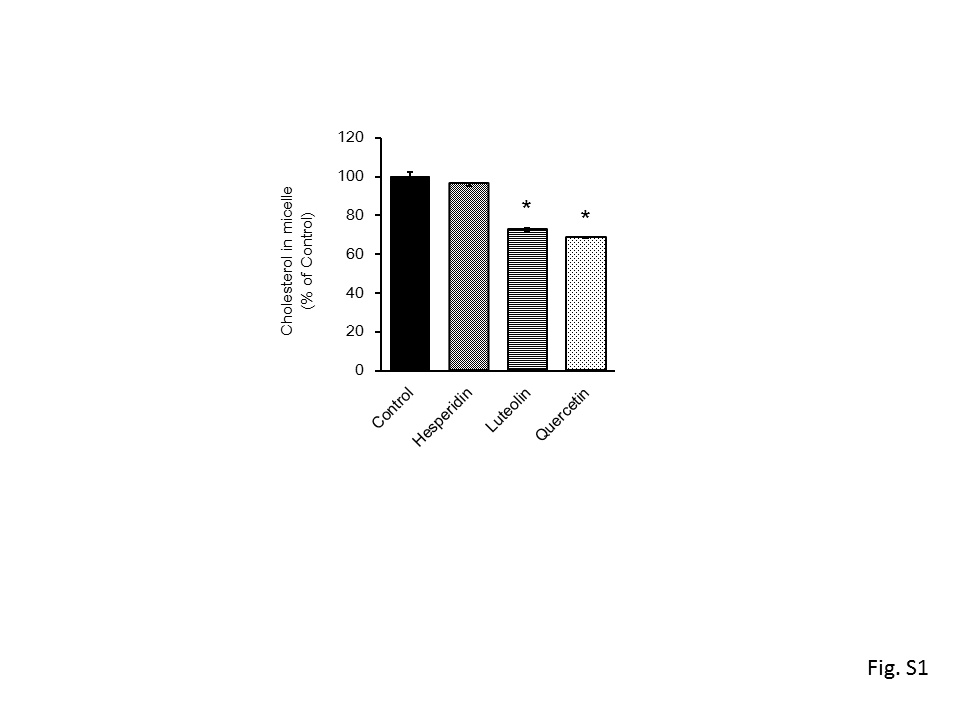

Supplement: Figure S1 — Inhibitory effects of luteolin and quercetin on micellar solubility of cholesterol. The effects of polyphenols on micellar solubility of cholesterol were assayed according to the method of Ikeda et al. [18], [19]. A bile salt micellar solution containing 4 mM sodium taurocholate, 0.1 mM egg yolk phosphatidylcholine (Sigma-Aldrich), 0.5 mM cholesterol, 0.3 nM [1,2-3H(N)]-cholesterol, and 1% (v/v) methanol was prepared by vortexing and stored at 37°C for at least 24 h. Polyphenols (final concentration: 1 mM each) were added to the micellar solution (100 µL) and maintained at 37°C for 1 h. The solution was passed through a 0.22-µm PVDF membrane filter (Ultrafree, Millipore), and the concentration of radioactive cholesterol in the filtrate was measured using a scintillation counter. Hesperidin, which did not significantly affect cholesterol uptake (Fig. 3), was used as a negative control. Values are mean ± standard error (n = 3). Statistical analyses were performed as in Fig. 1. (TIF) [file pone.0097901.s001.tif]

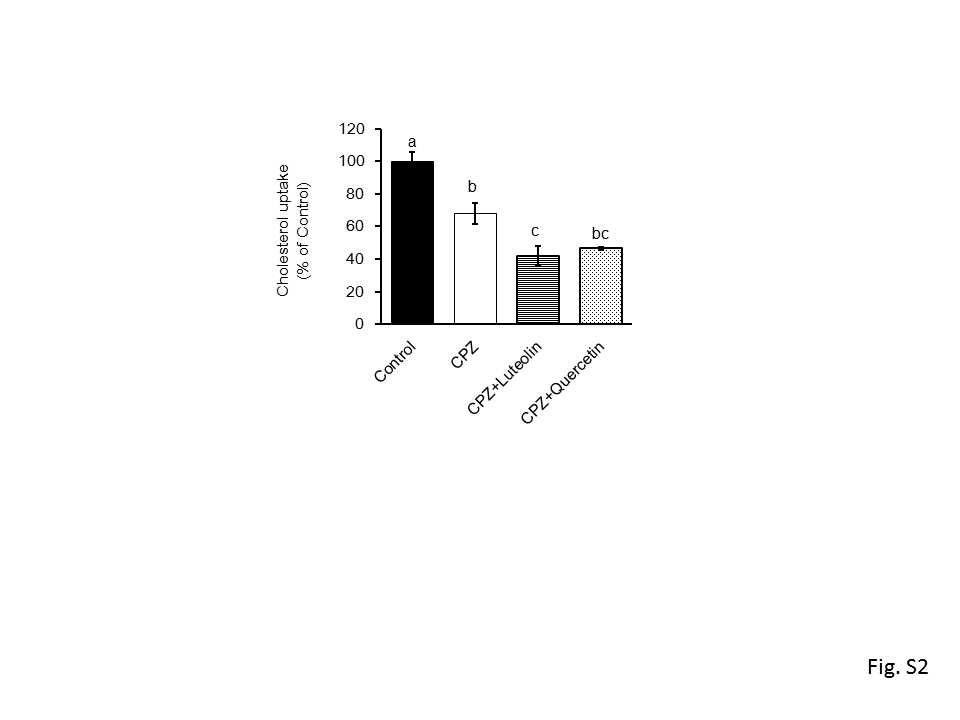

Supplement: Figure S2 — Inhibitory effects of simultaneously adding chlorpromazine (CPZ) with luteolin or quercetin. The Caco-2 cells were incubated in 50 µM CPZ (a clathrin-mediated endocytosis inhibitor) with or without 50 µM luteolin or quercetin at 37°C for 1 h, washed twice with HBSS (pH 7.4). The cholesterol micelle was then added, and uptake by Caco-2 cells was quantified. Values are mean ± standard error (n = 3). Statistical analyses were performed as described in the legend to Fig 5. (TIF) [file pone.0097901.s002.tif]
