# Supplementary material for: Luteolin and Quercetin Affect the Cholesterol Absorption Mediated by Epithelial Cholesterol Transporter Niemann–Pick C1-Like 1 in Caco-2 Cells and Rats
Source: PLoS One. 2014 May 23;9(5):e97901. doi: 10.1371/journal.pone.0097901 (PMC4032257; doi:10.1371/journal.pone.0097901)
Supplement: Table S1 — Composition of diets and oral administration of flavonoids. (DOCX) [file pone.0097901.s003.docx]

**Table S1**. Composition of diets and oral administration of flavonoids.

| Composition of diets (g/kg) | NC | HC | HL | HQ |
| --- | --- | --- | --- | --- |
| Cornstarch | 397.5 | 397.5 | 397.5 | 397.5 |
| Casein | 200.0 | 200.0 | 200.0 | 200.0 |
| Detrinized cornstarch | 132.0 | 132.0 | 132.0 | 132.0 |
| Sucrose | 102.5 | 102.5 | 102.5 | 102.5 |
| Soybean oil | 70.0 | 70.0 | 70.0 | 70.0 |
| Fiber | 50.0 | 50.0 | 50.0 | 50.0 |
| Mineral mix(AIN93G-MX) | 35.0 | 35.0 | 35.0 | 35.0 |
| Vitamin mix(AIN93G-VX) | 10.0 | 10.0 | 10.0 | 10.0 |
| L-cystine | 3.0 | 3.0 | 3.0 | 3.0 |
| Cholesterol |  | 5.0 | 5.0 | 5.0 |
| Oral administration (ml/kg) |  |  |  |  |
| 20 mM Luteolin (in 1% DMSO water) | |  | 5.0 |  |
| 20 mM Quercetin (in 1% DMSO water) |  |  |  | 5.0 |
| 1% DMSO water | 5.0 | 5.0 |  |  |
